# Overexpression of malic enzyme is involved in breast cancer growth and is correlated with poor prognosis

**DOI:** 10.1111/jcmm.18163

**Published:** 2024-03-06

**Authors:** Wan‐Chung Hu, Yi‐Fang Yang, Ching‐Feng Cheng, Ya‐Ting Tu, Hong‐Tai Chang, Kuo‐Wang Tsai

**Affiliations:** ^1^ Department of Clinical Pathology and Medical Research, Taipei Tzu Chi Hospital Buddhist Tzu Chi Medical Foundation New Taipei City Taiwan; ^2^ Department of Medical Education and Research Kaohsiung Veterans General Hospital Kaohsiung Taiwan; ^3^ Department of Pediatrics, Taipei Tzu Chi Hospital Buddhist Tzu Chi Medical Foundation New Taipei City Taiwan; ^4^ Institute of Biomedical Sciences, Academia Sinica Taipei Taiwan; ^5^ Department of Pediatrics Tzu Chi University Hualien Taiwan; ^6^ Department of Research Taipei Tzu Chi Hospital, Buddhist Tzu Chi Medical Foundation New Taipei City Taiwan; ^7^ Department of Surgery Kaohsiung Veterans General Hospital Kaohsiung Taiwan; ^8^ Department of Nursing Cardinal Tien Junior College of Healthcare and Management New Taipei City Taiwan

**Keywords:** breast cancer, malic enzyme, metabolism

## Abstract

Malic enzyme (*ME*) genes are key functional metabolic enzymes playing a crucial role in carcinogenesis. However, the detailed effects of *ME* gene expression on breast cancer progression remain unclear. Here, our results revealed *ME1* expression was significantly upregulated in breast cancer, especially in patients with oestrogen receptor/progesterone receptor‐negative and human epidermal growth factor receptor 2‐positive breast cancer. Furthermore, upregulation of *ME1* was significantly associated with more advanced pathological stages (*p* < 0.001), pT stage (*p* < 0.001) and tumour grade (*p* < 0.001). Kaplan–Meier analysis revealed *ME1* upregulation was associated with poor disease‐specific survival (DSS: *p* = 0.002) and disease‐free survival (DFS: *p* = 0.003). Multivariate Cox regression analysis revealed *ME1* upregulation was significantly correlated with poor DSS (adjusted hazard ratio [AHR] = 1.65; 95% CI: 1.08–2.52; *p* = 0.021) and DFS (AHR, 1.57; 95% CI: 1.03–2.41; *p* = 0.038). Stratification analysis indicated *ME1* upregulation was significantly associated with poor DSS (*p* = 0.039) and DFS (*p* = 0.038) in patients with non‐triple‐negative breast cancer (TNBC). However, *ME1* expression did not affect the DSS of patients with TNBC. Biological function analysis revealed *ME1* knockdown could significantly suppress the growth of breast cancer cells and influence its migration ability. Furthermore, the infiltration of immune cells was significantly reduced when they were co‐cultured with breast cancer cells with *ME1* knockdown. In summary, *ME1* plays an oncogenic role in the growth of breast cancer; it may serve as a potential biomarker of progression and constitute a therapeutic target in patients with breast cancer.

## 1 INTRODUCTION

Breast cancer occurs in mammary gland epithelial tissue and is the most frequently diagnosed cancer among women worldwide, with 99% of breast cancer cases occurring in women and only 1% occurring in men.^1^ The mammary gland may not be vital for sustaining life, and in situ breast cancer is not immediately fatal. However, the loose interconnection between breast cancer cells makes them prone to detachment. As a result, cancer metastasis can occur through the bloodstream and lymphatic system, posing a grave threat to a patient's life.^2^ Studies have revealed that invasive cancer cells are a primary factor contributing to breast cancer‐related deaths and are a major challenge in the treatment of breast cancer.^3^, ^4^ Therefore, investigations of additional markers that can predict treatment response, tumour advancement and potential targeted therapies have steadily increased.^5^

In the past two decades, physicians and cancer biologists have determined that at least four subtypes of breast cancer (i.e. luminal A, luminal B, human epidermal growth factor receptor 2 [HER2] and basal‐like) can be identified by their unique gene expression profiles.^6^ In Taiwan, the 5‐year survival rate of patients with early‐stage breast cancer is up to 90%, regardless of the molecular subtype^7^; however, a 10‐year follow‐up study indicated that patients with luminal type (A and B) breast cancer had a more favourable prognosis and a higher 5‐year survival rate than patients with HER2 type or triple‐negative breast cancer (TNBC).^7^, ^8^ Moreover, patients with TNBC had the poorest prognosis and the lowest 5‐year survival rate of all patients with breast cancer.^7^, ^8^ The poor prognosis of patients with breast cancer is strongly associated with metastasis and drug resistance. Metastasis accounts for 90% of cancer‐related deaths.^9^, ^10^, ^11^

### 1.1 Malic enzyme genes in human cancer

Malic enzyme (*ME*) genes are key functional metabolic enzymes, which can catalyse the conversion of malate to pyruvate, thus generating nicotinamide adenine dinucleotide phosphate (NADPH) from NADP+. Pyruvate is a primary substrate for the tricarboxylic acid cycle.^12^, ^13^ Studies have revealed that NADP‐dependent *ME* genes play a crucial role in maintaining cellular redox homeostasis and supporting energy in normal cells.^14^, ^15^ Three isoforms of *ME*s have been identified in mammalian cells: *ME1*, a cytosolic NADP+‐dependent isoform; *ME2*, a mitochondrial NAD+‐dependent isoform; and *ME3*, a mitochondrial NADP+‐dependent isoform.^16^, ^17^ Studies have revealed *ME* dysfunction in human cancer progression.^17^, ^18^, ^19^, ^20^ Lu et al. contended that *ME1* overexpression contributed to gastric cancer cell growth and metastasis by depleting NADPH and inducing high levels of reactive oxygen species (ROS).^21^ In oral cancer, the upregulation of *ME1* was closely associated with poor prognosis, and the knockdown of *ME1* expression inhibited cell proliferation and migration ability.^22^ Liao et al. asserted that *ME1* expression significantly promoted cancer cell growth and invasion in basal‐like breast cancer.^23^ However, the clinical effects of *ME1* in breast cancer and the detailed mechanisms underlying this relationship remain unclear. This study investigated the effects of *ME1* knockdown on breast cancer proliferation and migration using a human breast cancer cell line. The *ME1* expression levels in patients with breast cancer were also examined using a tissue microarray, which enabled an analysis of the relationship between clinicopathological features and *ME1* expression.

## 2 MATERIALS AND METHODS

### 2.1 Expression data from the Cancer Genome Atlas (TCGA)

We retrieved transcriptome data from 1079 breast cancer cases through The Cancer Genome Atlas (TCGA) data portal (https://tcga‐data.nci.nih.gov/tcga/dataAccessMatrix.htm). Clinical information for patients with breast cancer, encompassing gender, pathological stage and overall survival, was also acquired from TCGA. Utilizing TCGA data, we conducted an analysis to explore the clinical impacts of ME1 expression on both clinical pathological features and overall survival among breast cancer patients. To comprehensively assess the clinical impact of ME1 expression on the overall survival of breast cancer patients, we included additional cohorts and employed the Kaplan–Meier Plotter.^24^ Survival data were obtained from two sources: the Gene Expression Omnibus (GEO) (http://kmplot.com/analysis/index.php?p=service&cancer=breast), comprising a total of 1879 patients for overall survival analysis, and an RNA sequencing database (http://kmplot.com/analysis/index.php?p=service&cancer=breast_rnaseq_gse96058), including 2976 patients with breast cancer for this study's analysis.

### 2.2 In silico genetic analysis and *ME*1 in patients with breast cancer

Genetic variations in ME1 among breast cancer patients were examined using the cBioPortal for Cancer Genomics data set (cBioPortal, v.3.6.20) (http://www.cbioportal.org). This study utilized two data sources for analysing ME1 genetic variants in breast cancer: METABRIC (patient numbers: 2173)^25^, ^26^ and PanCancer Atlas (patient numbers: 1084).^27^ The impact of ME1 genetic variants on oestrogen receptor (ER) status, progesterone receptor (PR) status and histological grade in breast cancer patients was assessed through the cBioPortal.

### 2.3 Patients and tissues

This study received approval from the Institutional Review Board (IRB) of Kaohsiung Veterans General Hospital in Kaohsiung, Taiwan (IRB number: VGHKS13‐CT10‐10), and Taipei Tzu Chi Hospital in Taiwan (IRB number: 09‐XD‐154). Written informed consent was waived by the hospital IRB due to the utilization of previously collected and anonymized data and specimens. Tissue microarrays were employed to examine ME1 expression in this study. The tissue microarray comprised adjacent normal tissue (*n* = 483), ductal carcinoma in situ (DCIS) tissue (*n* = 215), invasive ductal carcinoma (IDC) (*n* = 497) and recurrent tissue (*n* = 27). These tissue specimens were collected from a total of 497 breast cancer patients.

### 2.4 Immunohistochemistry

IHC analysis was implemented using the Novolink Max Polymer Detection System (Product No: RE7280‐K, Leica, Newcastle Upon Tyne, United Kingdom). The slides underwent deparaffinization in xylene and were gradually rehydrated using alcohol. Antigen retrieval was implemented by subjecting the slides to tris‐ethylenediaminetetraacetic acid (10 mM, pH 9.0) at 125°C for 10 min in a pressure boiler. To block endogenous peroxidase activity, the slides were incubated with 3% hydrogen peroxide in methanol for 30 min. The slides were blocked with blocking buffer (RE7158) at room temperature, primary antibodies were applied, and the slides were then incubated overnight at 4°C in a humid chamber. The primary antibody used in this study was rabbit polyclonal anti‐*ME1* (1:100; H00004199‐M03, Abnova) in Tris‐buffered saline solution with 5% bovine serum albumin. Secondary antibodies were used from the Novolink Max Polymer Detection System (RE7280‐K, Leica, Newcastle Upon Tyne, United Kingdom) in accordance with the manufacturer's instructions. The slides were rinsed with phosphate‐buffered saline and incubated with secondary antibody according to the manufacturer's protocol. The slides were incubated with Post Primary (RE7159) for 10 min at room temperature. Then the slides were rinsed with phosphate‐buffered saline and incubated with Novolink Polymer (RE7161) for 10 min at room temperature. Furthermore, the slides developed peroxidase activity with DAB working solution (RE7162 and RE7163) and counterstained with haematoxylin (RE7164).

### 2.5 Immunohistochemistry analysis and scoring

Initially, a senior pathologist and a technician jointly assessed the slides until all discrepancies were resolved. Subsequently, the technician independently reviewed all the slides. Finally, a random selection of 5%–20% of core samples at each intensity was re‐evaluated by the pathologist. Throughout the evaluation process, both the pathologist and technician remained blinded to the clinical outcomes of the patients. Immunoreactivity was graded using a semiquantitative approach. Marker scores were determined on the basis of staining intensity (i.e. 0: no signal, 1: mild, 2: moderate and 3: strong) and the proportion of positively stained tumour cells in five high‐power fields (i.e. 0: <5%, 1: 5%–25%, 2: 26%–50%, 3: 51%–75% and 4: >75%). The marker score indicated the sum of the staining intensity score and the percentage of positively stained tumour cells. The overall score was categorized as follows: – (0–1), + (2, 3), ++ (4, 5) and +++ (6, 7).

### 2.6 Cell line

Eight human cell lines, MCF‐7, T‐47D, SK‐BR‐3, BT‐549, Hs578T, MDA‐MB‐231, MDA‐MB‐453 and MDA‐MB‐468, were obtained from the American Type Culture Collection (ATCC) and cultured in Dulbecco's modified Eagle's medium (DMEM) supplemented with 10% inactivated fetal bovine serum (FBS; Invitrogen, Carlsbad, CA, USA). The highly metastatic MDA‐MB‐231‐IV2‐1 cells were generously provided by Dr. Lu‐Hai Wang, with detailed information available in previous study.^28^ The THP‐1 cell line was sourced from ATCC and maintained in Roswell Park Memorial Institute (RPMI) 1640 medium. This medium was adjusted to contain 2 mM L‐glutamine, 1.5 g/L sodium bicarbonate, 4.5 g/L glucose, 10 mM HEPES and 1.0 mM sodium pyruvate, and supplemented with 0.05 mM 2‐mercaptoethanol and 10% inactivated FBS.

### 2.7 Stable *ME1* knockdown with shRNA

Breast cancer cells, Hs578t, MDA‐MB‐231 and MCF‐7, were seeded in a 6 cm culture dish at a density of 2.5 × 10^5^ cells/mL. Then, stable breast cancer cells with *ME1* knockdown were generated by infecting breast cancer cells with lentiviruses expressing sh*ME1* in the presence of 8 μg/mL of polybrene for 24 h. Puromycin (4 μg/mL) selection was then applied for 3–5 days. The sh‐luciferase vector, targeting the luciferase gene and providing puromycin resistance, was used as the control. *ME1* expression was verified through western blotting.

### 2.8 Western blotting

Cell lysates were obtained using a radioimmunoprecipitation assay buffer (50 mM Tris–HCl at pH 8.0, 150 mM NaCl, 1% NP‐40, 0.5% deoxycholic acid and 0.1% sodium dodecyl sulfate). The total proteins were then separated using 6%–10% sodium dodecyl sulfate–polyacrylamide gel electrophoresis and transferred to nitrocellulose filter membranes (Millipore, Billerica, USA). Subsequently, the membranes were blocked with a blocking buffer at room temperature for 1 h. The membranes were then incubated overnight at 4°C with the primary antibody (rabbit polyclonal anti‐*ME1* at a dilution of 1:100; H00004199‐M03, Abnova). After three washes with Tris‐buffered saline containing Tween‐20 buffer (50 mM Tris–HCl at a pH of 7.6, 150 mM NaCl and 0.1% Tween‐20), the membranes were then treated with a horseradish peroxidase‐conjugated secondary antibody (1:10,000, Santa Cruz Biotechnology, Inc.) at room temperature for 1 h. Finally, the proteins were visualized using WesternBright ECL reagent (Advansta, Menlo Park, CA, USA) and were detected with the BioSpectrumTW 500 Imaging System (UVP, USA).

### 2.9 Cell proliferation assays

Three breast cancer cells, Hs578T, MDA‐MB‐231 and MCF‐7, with ME1 knockdown or control were seeded in a 96‐well plate at a density of 2.5 × 10^3^ cells/mL. The growth of the cells was assessed at 0, 1, 2, 3 and 4 days using the CellTiter‐Glo One Solution Assay (Promega, Madison, WI, USA). All experiments were conducted in triplicate.

### 2.10 Colony formation ability assay

A total of 4000 breast cancer cells (Hs578T, MDA‐MB‐231 and MCF‐7) with ME1 knockdown or control were plated into a 6‐well plate and then incubated at 37°C for 2 weeks. Then, the culture plates containing colonies of breast cancer cells were fixed with 3.7% formaldehyde for 10 min, and colonies were stained with crystal violet. Then, the relative colony formation ability was measured on a spectrophotometer at a wavelength of 620 nm. All experiments were conducted in triplicate.

### 2.11 Invasion assays

The invasion ability of breast cancer cells was assessed in vitro by employing a transwell assay, as described in our previous study.^29^ In summary, total of 3 × 10^5^ breast cancer cells (Hs578T or MDA‐MB‐231) with ME1 knockdown or a scrambled control were placed in a suspension containing 2% FBS. These cells were then seeded onto the upper chamber of Falcon transwells (Falcon, Corning, USA), which were coated with Matrigel (BD Biosciences, MA, USA) to facilitate the invasion assay. Subsequently, the cells were placed in a CO_2_ incubator at 37°C for either 12 or 24 h. After the incubation period, any remaining cells in the upper chamber were removed using cotton swabs, whereas cells on the undersurface of the transwells were fixed using a 10% formaldehyde solution. The cells were then stained with crystal violet solution, and the number of breast cancer cells was determined by counting the three fields with a phase‐contrast microscope. Each experiment was completed three times to ensure accuracy.

### 2.12 Analysis of macrophage infiltration

We analysed the correlations between *ME1* gene expression and the distribution of human immune cells in breast tumours by employing **T**umor **Im**mune **E**stimation **R**esource 2.0 (TIMER2.0; http://timer.cistrome.org/),^30^ functions as a platform for systematically analysing the immunological characteristics of cancer according to information of The Cancer Genome Atlas (TCGA). In this study, we evaluated the correlations between *ME1* expression and the infiltration of B cells, CD8+ T cells, CD4+ T cells, macrophages, neutrophils and dendritic cells. Correlations were calculated using Spearman's rho value, and scatterplots were used.

### 2.13 Macrophage‐induced breast cancer cells migration

MDA‐MB‐231 (3 × 10^5^) with ME1 knockdown or a scrambled control were placed in a DMEM medium supplemented with 2% inactivated FBS (Invitrogen, Carlsbad, CA, USA). These cells were then seeded onto the upper chamber of Falcon transwells (Falcon, Corning, USA) for migration assay. THP‐1 cells were induced macrophage by PAM, then THP‐1‐induced macrophage cells (8 × 10^4^) were seeded in the lower chamber containing RPMI 1640 growth medium as described above. Subsequently, the cells were placed in a CO_2_ incubator at 37°C for 7 h. After the incubation period, any remaining cells in the upper chamber were removed using cotton swabs, whereas cells on the undersurface of the transwells were fixed using a 10% formaldehyde solution. The cells were then stained with crystal violet solution, and the number of breast cancer cells was determined by counting three fields with a phase‐contrast microscope. Each experiment was completed three times to ensure accuracy.

### 2.14 Macrophage infiltration assay

For macrophage cell infiltration, THP‐1 cells were induced macrophage by PAM, then THP‐1‐induced macrophage cells (5 × 10^5^) were seeded in the upper chamber of Falcon transwells (Falcon, Corning, USA) containing RPMI 1640 medium without FBS. MDA‐MB‐231 cells (8 × 10^4^) with ME1 knockdown or a scrambled control were placed in lower chamber containing DMEM medium supplemented with 10% FBS. Subsequently, the cells were placed in a CO_2_ incubator at 37°C for 24 h. After the incubation period, any remaining cells in the upper chamber were removed using cotton swabs, whereas cells on the undersurface of the transwells were fixed using a 10% formaldehyde solution. The cells were then stained with crystal violet solution, and the number of macrophage cells was determined by counting three fields with a phase‐contrast microscope. Each experiment was completed three times to ensure accuracy.

### 2.15 Statistical analysis

Several statistical methods were employed for data analysis. Correlations between protein expression levels and types of breast tissues or clinicopathological parameters were assessed using various tests, such as the chi‐squared test, Student's *t*‐test, analysis of variance (anova), the Mann–Whitney *U* test and the Kruskal–Wallis one‐way anova. In studies related to breast cancer, the outcomes were generally defined as the time from diagnosis or surgery to a specific event of interest (i.e. the end point). DSS was measured from the time of initial resection of the primary tumour to the date of cancer‐related death or last follow‐up. DFS was defined as the time from surgery to an event such as local recurrence, regional recurrence or distant metastasis, excluding disease‐related death. Cumulative survival curves were estimated using the Kaplan–Meier method, and the log‐rank test was implemented to compare the relevant survival curves. This study employed a Cox proportional hazards model to identify independent predictors of survival, incorporating significant factors from the univariate analysis as covariates. Statistical significance was indicated by a two‐tailed *p* < 0.05. All statistical analyses were performed using SPSS version 20.0 for Windows (SPSS Inc., Armonk, NY, USA).

## 3 RESULTS

### 3.1 *ME1* was deregulated in breast cancer

*ME*s are crucial metabolic enzymes that play a vital role in supporting cellular energy and redox balance and are essential for physiological functions within cells. Studies have revealed that *ME* dysfunction crucially affects cancer progression by altering metabolic reprogramming and redox homeostasis in human cancer.^14^, ^20^, ^31^, ^32^ To investigate how abnormalities in *ME* genes affect breast cancer, this study first implemented an in silico analysis using cBioPortal to examine genetic variations in *ME1*. The results revealed that the percentage of genetically altered *ME1* was 1.1 in breast cancer (Figure S1A). Among the genetic variations, the most frequent event in human breast cancer was the genomic amplification of *ME*s. As indicated in Figure S1B, *ME1* had a 1.0% amplification ratio (31 out of 3257) in patients with breast cancer. Furthermore, the occurrence of genetic variations in *ME1* was significantly associated with poor histological grade (*p* = 4.76E^−4^). Notably, the results indicated a positive association between the patients with genetic variations in *ME1* and oestrogen receptor‐negative (ER‐) and progesterone receptor‐negative (PR‐) breast cancer (ER status: *p* = 3.693E^−4^ and PR status: *p* = 1.804E^−4^; Figure S1D,E). These results implied that genetic variations in *ME1* may be associated with poor prognosis and ER and PR status.

**FIGURE 1:**
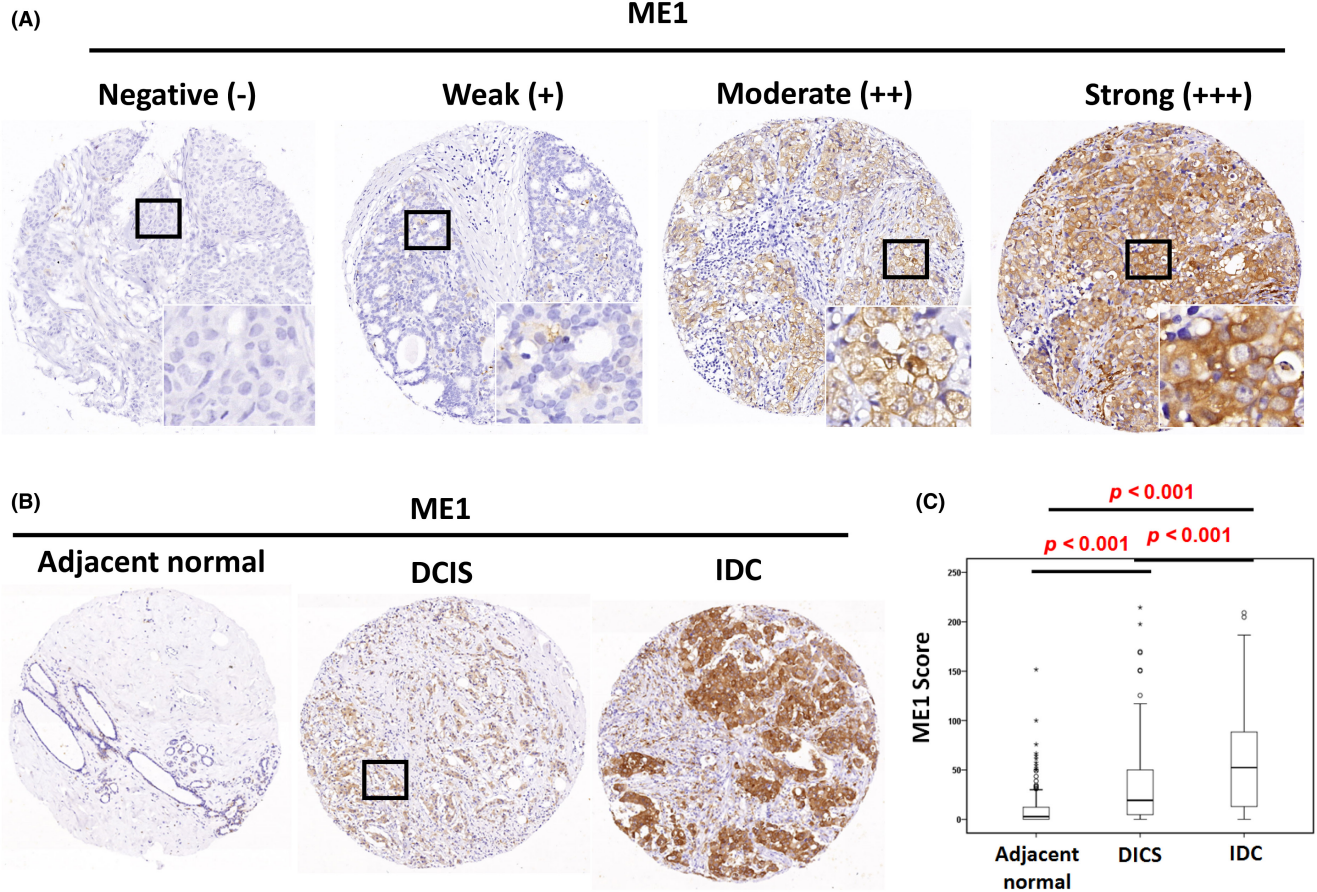
*ME1* expression was significantly upregulated in breast cancer. (A) Protein levels of *ME1* in breast cancer were examined in tissue microarrays containing samples from 497 patients using IHC. Photomicrographs indicated negative (−), weak (+), moderate (++) and strong (+++) staining in IDC tissues. (B) Photomicrographs indicated that *ME1* expression was upregulated in DCIS and IDC tissues compared with corresponding adjacent normal tissues. (C) *ME1* was significantly upregulated in DCIS and IDC tissues compared with adjacent normal tissues.

The results of the in silico analysis implied that *ME1* deregulation may contribute to poor prognosis in patients with breast cancer. To analyse the clinical effects of *ME1* in breast cancer, we implemented an IHC analysis to assess the protein expression levels of *ME1* in tissue microarrays containing adjacent normal tissues (*n* = 483), DCIS tissues (*n* = 215), IDC tissues (*n* = 497) and tissues with recurrence (*n* = 27). The samples were obtained from patients with breast cancer. The results revealed that *ME1* was expressed in the cytoplasm (Figure 1A), and *ME1* expression levels were significantly higher in the DCIS tissues (38.96 ± 50.45; *p* < 0.001) and IDC tissues (57.05 ± 56.09; *p* < 0.001;) than in adjacent normal tissues (15.87 ± 35.54, Table 1 and Figure 1B). In addition, the expression level of *ME1* was highest in the IDC tissues of patients with cancer recurrence (80.45 ± 63.17; *p* < 0.001). Therefore, the protein expression levels of *ME1* gradually increased during breast cancer progression from adjacent normal tissues or DCIS tissues.

**TABLE 1:** *ME1* expression in four tissue samples of IDC.

Adjacent normal		DCIS		IDC		Recurrence		*χ* ^2^	*p*‐value*
Mean ± SD	Median	Mean ± SD	Median	Mean ± SD	Median	Mean ± SD	Median		
(*n* = 483)		(*n* = 215)		(*n* = 497)		(*n* = 27)			
15.87 ± 35.54^a^, ^e^, ^f^, ^g^	1.19	38.96 ± 50.45^b^, ^e^, ^h^, ^i^	17.45	57.05 ± 56.09^c^, ^f^, ^h^	39.68	80.45 ± 63.17^d^, ^g^, ^i^	94.00	282.06	<0.001

Abbreviation: SD, standard deviation.

* *p*‐values were estimated by Kruskal–Wallis one‐way anova test.

^a^ *p* < 0.001;

^b^ *p* = 0.032;

^c^ *p* < 0.001;

^d^ *p* < 0.001;

^e^ *p* < 0.001;

^f^ *p* < 0.001;

^g^ *p* < 0.001;

^h^ *p* < 0.001;

^i^ *p* = 0.001.

### 3.2 High *ME1* expression levels were associated with poor clinicopathological features

**FIGURE 2:**
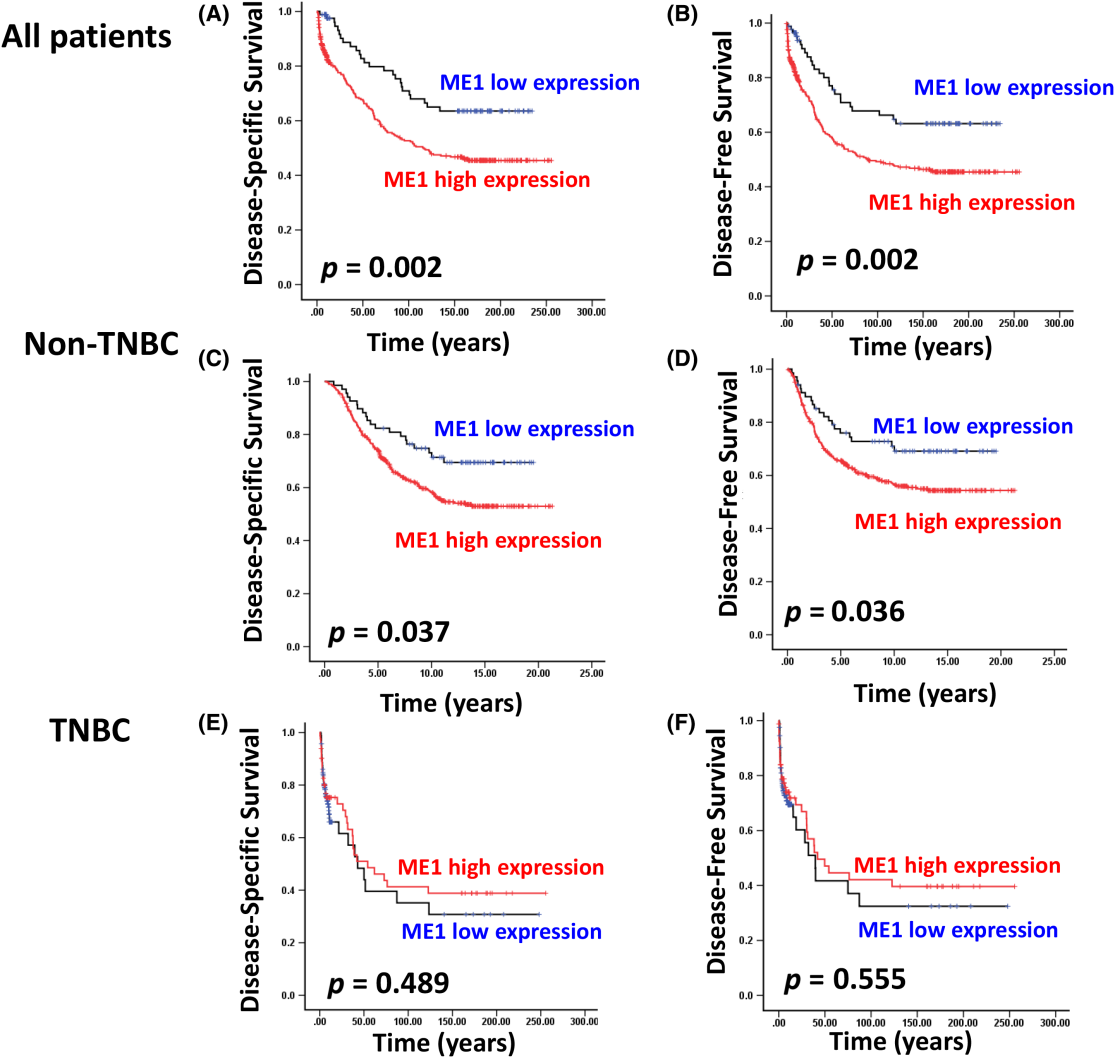
*ME1* expression level was significantly correlated with the survival curve of patients with breast cancer. (A and B) DSS and DFS were compared with respect to *ME1* expression in breast cancer using a log‐rank test. (C and D) DSS and DFS were compared with respect to *ME1* expression in non‐TNBC using a log‐rank test. (E and F) DSS and DFS were compared with respect to *ME1* expression in TNBC using a log‐rank test.

We investigated whether *ME1* protein dysfunction is associated with the clinicopathological features of breast cancer. Our data revealed that *ME1* upregulation was significantly correlated with advanced pathological stage (*p* < 0.001), pT stage (*p* < 0.001) and tumour grade (*p* < 0.001; Table 2). A survival analysis revealed that *ME1* upregulation was significantly associated with poor disease‐specific survival (DSS: crude HR [CHR] = 1.93, 95% CI: 1.27–2.95, *p* = 0.002) and disease‐free survival (DFS: CHR = 1 0.90, 95% CI: 1.25–2.90, *p* = 0.003; Table 3 and Figure 2A,B). Multivariate logistic analysis revealed that *ME1* upregulation was significantly associated with DSS (adjusted HR [AHR] = 1.65, 95% CI: 1.08–2.52, *p* = 0.021) and DFS (AHR = 1.57, 95% CI: 1.03–2.41, *p* = 0.038) in patients with breast cancer (Table 3). An analysis of a public database verified these findings. Data on the clinical pathological features and *ME1* expression levels of 1079 patients with breast cancer were downloaded from TCGA database. As presented in Table S1, *ME1* expression was significantly upregulated in patients with an advanced pathological stage (*p* = 0.047). We next examined the relationship between *ME1* expression and the postoperative survival of patients with breast cancer. Univariate Cox regression analysis revealed that *ME1* upregulation was correlated with poor survival outcomes (CHR = 2.18, 95% CI: 1.38–3.43, *p* = 0.001; Table S2). Multivariate Cox regression analysis revealed that *ME1* upregulation was an independent prognostic biomarker of overall survival in patients with breast cancer (AHR = 2.10, 95% CI: 1.32–3.34, *p* = 0.002; Table S2). The analysis of the Gene Expression Omnibus (GEO database) and an RNA sequencing database revealed an association between *ME1* upregulation and poor survival curves in patients with breast cancer (Figure S2A,B). Overall, the results revealed that *ME1* upregulation was associated with poor prognosis in patients with breast cancer.

**TABLE 2:** Correlation of *ME1* expression with clinicopathological characteristics of patients with IDC.

Variables	ME1 (*n* = 497)			
	%	Mean ± SD	Median	*p*‐value
Age (year)				
<40	14.3	53.72 ± 48.41	50.47	0.371^b^
40–59	57.7	54.19 ± 53.77	35.90	
>60	28.0	64.65 ± 63.60	46.01	
BMI				
Underweight + normal	50.1	54.99 ± 54.13	38.54	0.721^b^
Overweight	28.8	62.40 ± 61.99	38.81	
Obesity	21.1	54.65 ± 52.11	40.28	
Menopausal status				
Peri‐ and pre‐menopausal	49.1	57.47 ± 53.86	47.04	0.871^c^
Post‐menopausal	50.9	56.65 ± 58.26	33.33	
Pathology stage				
I	19.5	38.77 ± 47.22^d^, ^e^	13.83	<0.001^b^
II	49.9	63.03 ± 59.10^d^	50.42	
III	30.6	58.96 ± 54.10^e^	44.72	
pT stage				
T1	30.0	42.58 ± 48.96^f^, ^g^	20.29	<0.001^b^
T2	60.4	61.36 ± 56.52^f^	49.52	
T3 + T4	9.6	75.04 ± 64.95^g^	64.12	
pN stage				
N0	46.9	54.70 ± 55.70	35.98	0.720^a^
N1	25.6	60.11 ± 59.07	45.00	
N2	17.9	60.71 ± 54.33	46.16	
N3	9.6	53.63 ± 54.00	33.72	
Grading				
Well + moderate	62.0	47.82 ± 55.48	22.82	<0.001^c^
Poor	38.0	72.10 ± 53.91	60.94	
Vascular invasion (*n* = 495)				
Absent	61.2	55.53 ± 57.32	35.90	0.462^c^
Present	38.8	59.33 ± 54.13	45.72	
Nipple invasion				
Absent	93.4	56.74 ± 55.89	39.98	0.638^c^
Present	6.6	61.50 ± 59.47	33.33	

^a^ *p*‐values were estimated by one‐way anova test.

^b^ *p*‐values were estimated by Kruskal–Wallis one‐way anova test.

^c^ *p*‐values were estimated by student's *t*‐test.

^d^ *p* < 0.001;

^e^ *p* = 0.001;

^f^ *p* < 0.001;

^g^ *p* = 0.001.

**TABLE 3:** Univariate and multivariate Cox regression analyses of *ME1* expression for DSS and DFS in patients with IDC.

Characteristics	No. (%)	DSS				DFS			
		CHR^a^ (95% CI)	*p*‐value	AHR^b^ (95% CI)	*p*‐value	CHR^a^ (95% CI)	*p*‐value	AHR^b^ (95% CI)	*p*‐value
ME1	(*n* = 497)								
Low	85 (17.1)	1.00		1.00		1.00		1.00	
High	412 (82.9)	1.93 (1.27–2.95)	0.002	1.65 (1.08–2.52)	0.021	1.90 (1.25–2.90)	0.003	1.57 (1.03–2.41)	0.038

Abbreviations: AHR, adjusted hazard ratio; CHR, crude hazard ratio; DFS, disease‐free survival; DSS, disease‐specific survival.

^a^ CHR were estimated by univariate Cox's regression.

^b^ AHR were adjusted for AJCC pathological stage (II and III vs I), grading (III vs I and II), incomplete or inappropriate adjuvant treatment versus non‐treatment or complete adjuvant treatment and molecular subtypes (luminal B, Her2 over‐expression and basal‐like vs luminal A) by multivariate Cox's regression.

### 3.3 *ME1* expression was significantly correlated with certain breast cancer subtypes

**FIGURE 3:**
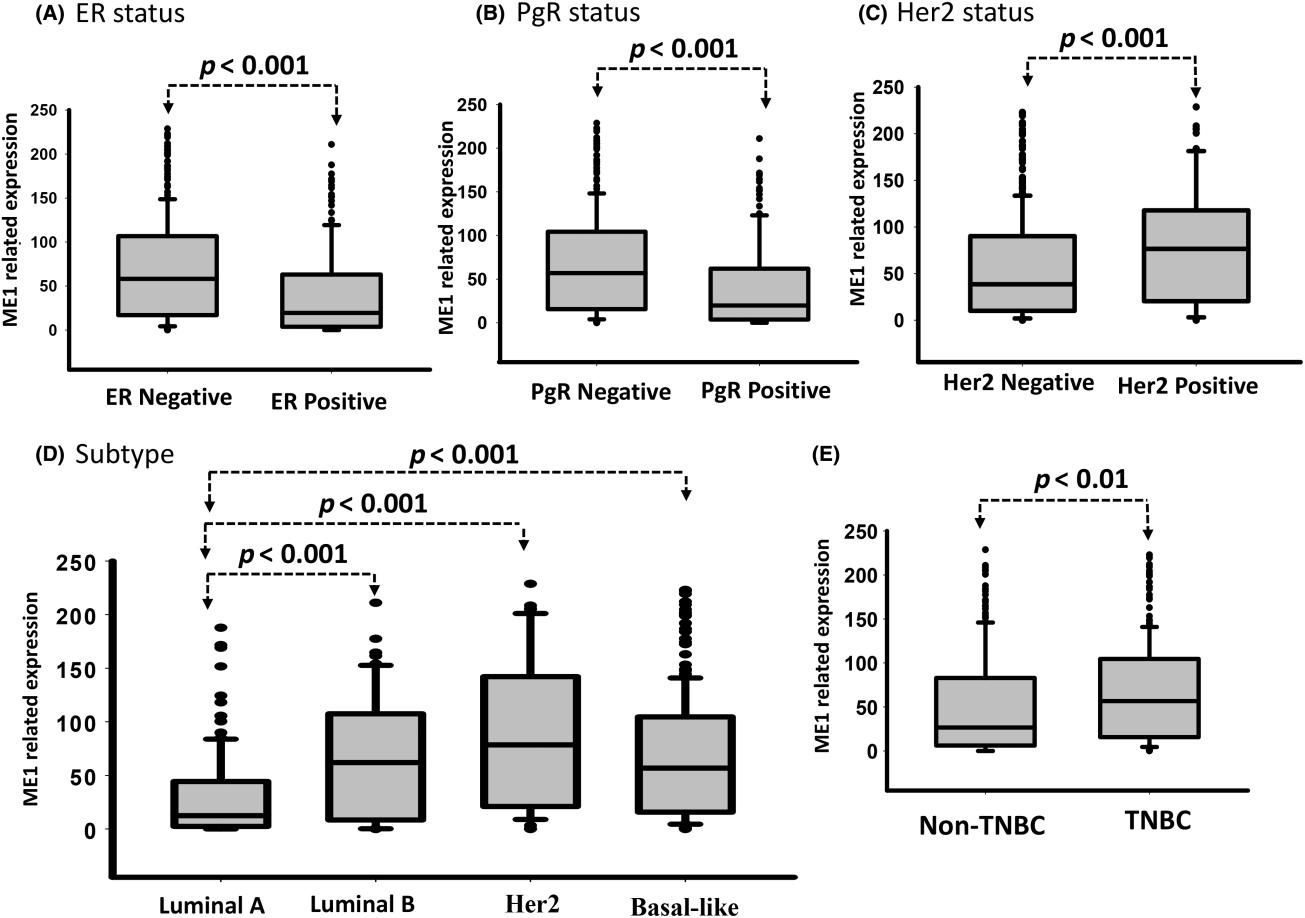
*ME1* expression in breast cancer with different molecular types. Expression level of *ME1* proteins was examined in molecular subtypes, such as by (A) ER status, (B) PR status and (C) HER2 status; (D) comparison between luminal A, luminal B, HER2 and basal‐like subtypes; (E) comparison between non‐TNBC and TNBC using IHC.

Notably, our data indicated that *ME1* high expression was significantly correlated with ER‐negative (*p* < 0.001), PR‐negative (*p* < 0.001) and HER2‐positive (*p* = 0.001; Figure 3A–C and Table 4) subtypes. *ME1* upregulation was significantly higher in the patients with IDC with the TNBC subtype (ER− PR− HER2−), luminal B subtype (ER+ PR+ HER2+) and HER2‐positive subtype (ER−/PR−/HER2+) compared with those with the luminal A subtype (ER+ PR+ HER2−; Table 4 and Figure 3D). We further analysed the correlation between *ME1* expression and clinical pathological features in the non‐TNBC and TNBC groups. As presented in Figure 3E, *ME1* expression was significantly upregulated in the TNBC group compared with the non‐TNBC group. Furthermore, high *ME1* expression levels were significantly correlated with advanced pathological stage (*p* = 0.004), pT stage (*p* = 0.005) and tumour grade (*p* = 0.005) in patients with non‐TNBC (Table S3). Similarly, *ME1* upregulation was associated with large tumour size (*p* = 0.013) and poor tumour grade (*p* = 0.002) in patients with TNBC (Table S4). Stratification analysis revealed a significant association between *ME1* upregulation and poor DSS (CHR = 1.68, 95% CI: 1.03–2.73, *p* = 0.039) and DFS (CHR = 1.68, 95% CI: 1.03–2.74 *p* = 0.038) in patients with non‐TNBC (Table 5 and Figure 2C,D). Notably, the results did not indicate a significant correlation between *ME1* expression and DSS (CHR = 0.85, 95% CI: 0.54–1.34, *p* = 0.490) and DFS (CHR = 0.87, 95% CI:0.56–1.37, *p* = 0.555) in patients with TNBC (Table 6 and Figure 2E,F). Our results revealed that *ME1* upregulation was associated with poor prognosis, and *ME1* expression could be used to classify breast cancer into molecular subtypes. These data suggested that *ME1* may be involved in the growth of breast cancer cells, and *ME1* upregulation was a biomarker of poor outcomes in patients with breast cancer, especially in patients with non‐TNBC.

**TABLE 4:** Correlation of *ME1* expression with molecular markers for IDC.

Variables	ME1			
	*n*	Mean ± SD	Median	*p*‐value
	%			
ER status	(*n* = 472)			
Negative	64.2	68.83 ± 57.91	58.18	<0.001^b^
Positive	35.8	40.83 ± 49.30	18.14	
PgR status	(*n* = 481)			
Negative	68.0	67.57 ± 57.35	56.68	<0.001^b^
Positive	32.0	38.53 ± 48.47	17.24	
Her2 status	(*n* = 487)			
Negative	85.4	54.15 ± 54.25	35.47	0.001^a^
Positive	14.6	78.93 ± 63.03	67.33	
Molecular subtype	(*n* = 464)			
Luminal A	25.0	28.75 ± 39.53^d^, ^e^, ^f^	12.27	<0.001^c^
Luminal B	11.9	66.08 ± 56.98^d^	60.87	
Her2	10.3	85.21 ± 67.81^e^	78.42	
Basal‐like	52.8	66.38 ± 55.78^f^	56.68	

^a^ *p*‐values were estimated by student *t*‐test.

^b^ *p*‐values were estimated by Mann–Whitney *U* test.

^c^ *p*‐values were estimated by Kruskal‐Wallis one‐way anova test.

^d^ *p* < .001;

^e^ *p* < 0.001;

^f^ *p* < 0.001.

**TABLE 5:** Univariate and multivariate Cox regression analyses of *ME1* expression for DSS and DFS in patients with non‐TNBC.

Characteristics	No. (%)	DSS				DFS			
		CHR^a^ (95% CI)	*p*‐value	AHR^b^ (95% CI)	*p*‐value	CHR^a^ (95% CI)	*p*‐value	AHR^b^ (95% CI)	*p*‐value
ME1	(*n* = 243)								
Low	64 (26.3)	1.00		1.00		1.00		1.00	
High	179 (73.7)	1.68 (1.03–2.73)	0.039	1.41 (0.86–2.31)	0.172	1.68 (1.03–2.74)	0.038	1.35 (0.82–2.21)	0.236

Abbreviations: AHR, adjusted hazard ratio; CHR, crude hazard ratio; DFS, disease‐free survival; DSS, disease‐specific survival.

^a^ CHR were estimated by univariate Cox's regression.

^b^ AHR were adjusted for AJCC pathological stage (II and III vs I), grading (III vs I and II), incomplete or inappropriate adjuvant treatment versus non‐treatment or complete adjuvant treatment and molecular subtypes (luminal B, Her2 over‐expression and basal‐like vs luminal A) by multivariate Cox's regression.

**TABLE 6:** Univariate and multivariate Cox regression analyses of *ME1* expression for DSS and DFS in patients with TNBC.

Characteristics	No. (%)	DSS				DFS			
		CHR^a^ (95% CI)	*p*‐value	AHR^b^ (95% CI)	*p*‐value	CHR^a^ (95% CI)	*p*‐value	AHR^b^ (95% CI)	*p*‐value
ME1	(*n* = 247)								
Low	165 (66.8)	1.00		1.00		1.00		1.00	
High	82 (33.2)	0.85 (0.54–1.34)	0.490	0.79 (0.50–1.24)	0.303	0.87 (0.56–1.37)	0.555	0.81 (0.51–1.27)	0.354

Abbreviations: AHR, adjusted hazard ratio; CHR, crude hazard ratio; DFS, disease‐free survival; DSS, disease‐specific survival.

^a^ CHR were estimated by univariate Cox's regression.

^b^ AHR were adjusted for AJCC pathological stage (II and III vs I), grading (III vs I and II), incomplete or inappropriate adjuvant treatment versus non‐treatment or complete adjuvant treatment and molecular subtypes (luminal B, Her2 over‐expression and basal‐like vs luminal A) by multivariate Cox's regression.

### 3.4 *ME1* expression contributed to breast cancer cell growth

**FIGURE 4:**
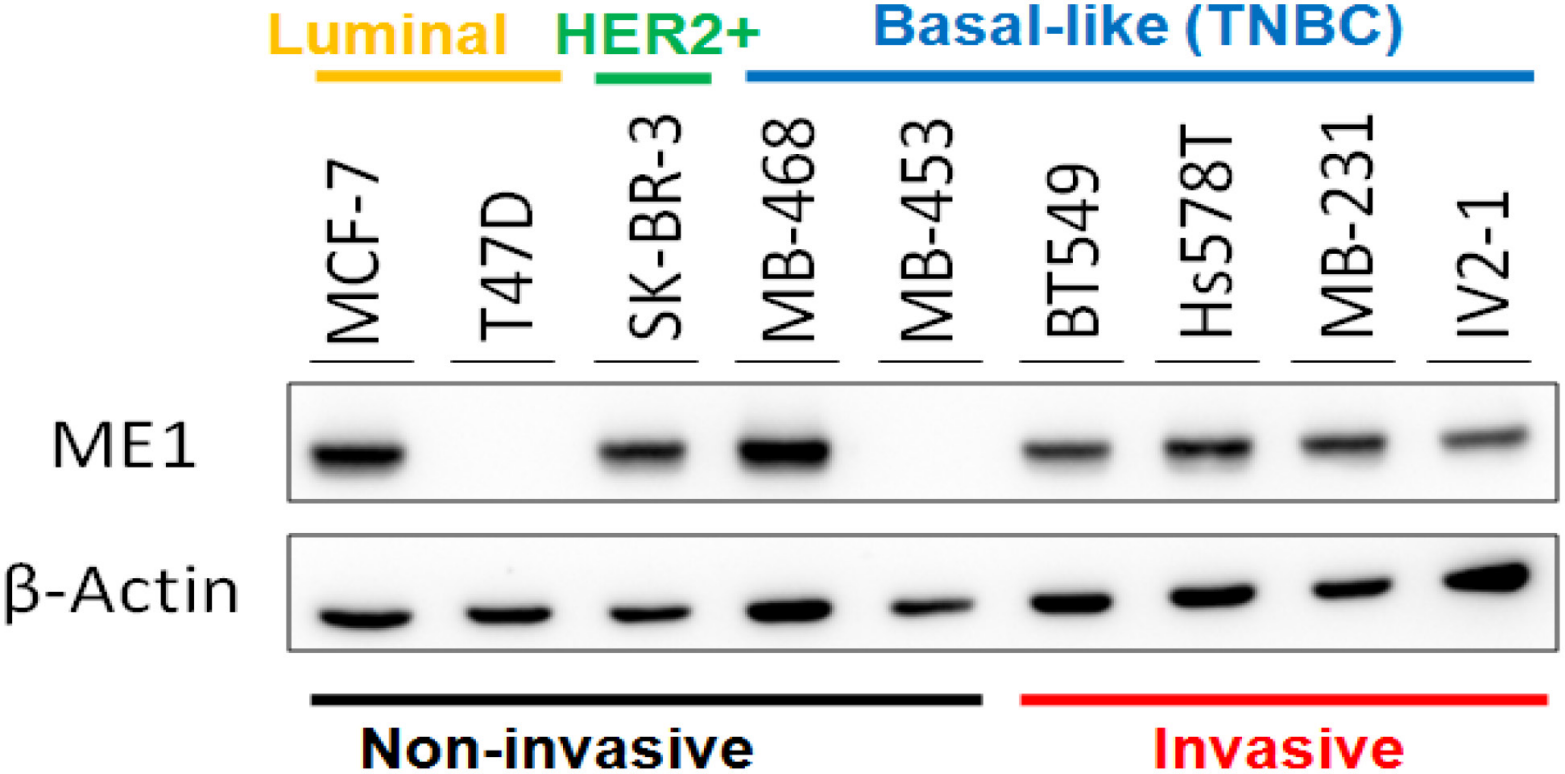
Expression levels of *ME1* were examined in breast cancer cells using western blotting.

**FIGURE 5:**
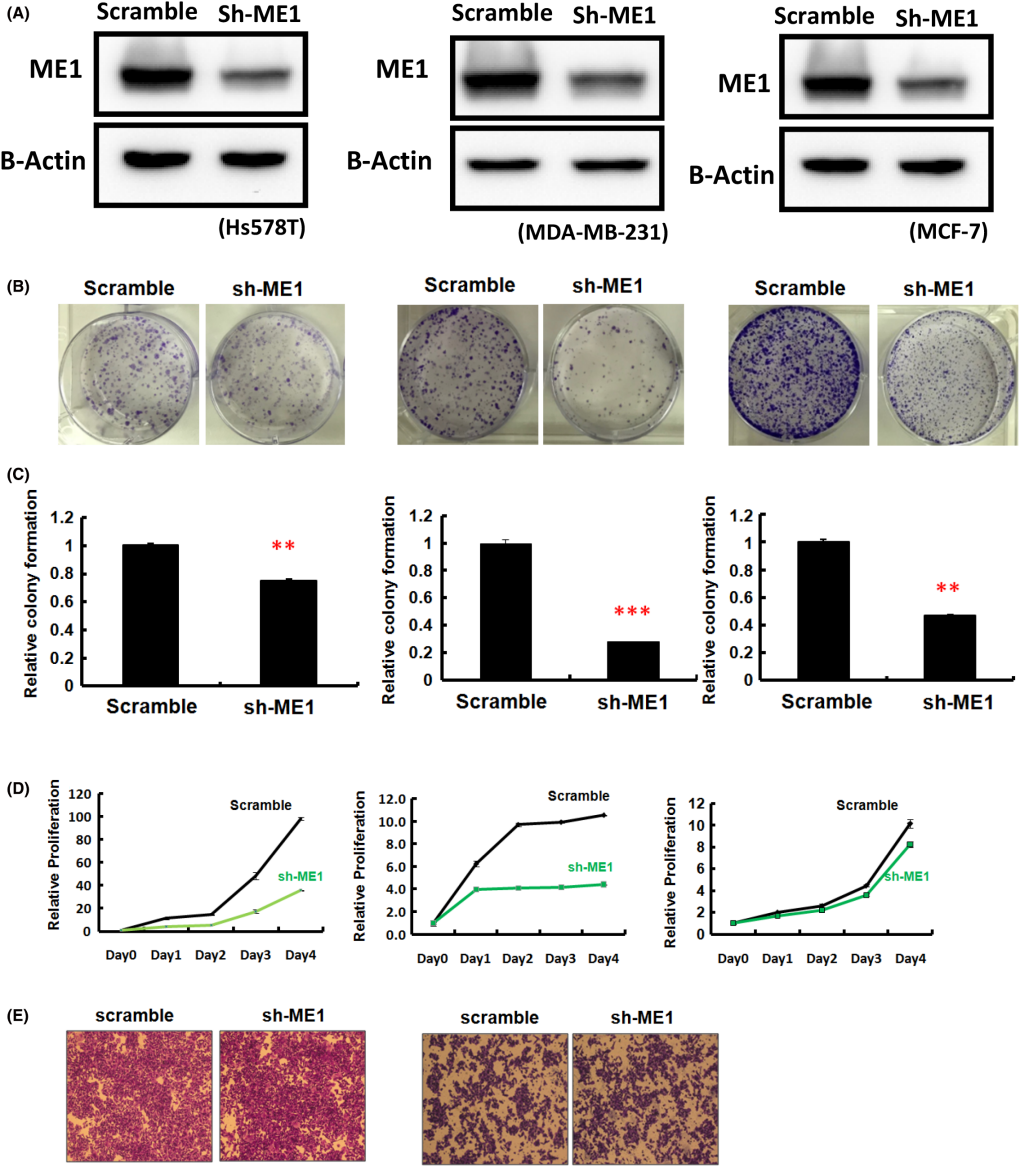
Examination of cellular function of *ME1* in breast cancer cell lines. First, shRNAs of *ME1* were individually transfected into breast cancer cells (Hs578T, MDA‐MB‐231 and MCF7). (A) Relative expression of *ME1* was examined in breast cancer cells after shRNA transfection using western blotting. (B and C) Colony formation assay after *ME1* knockdown. (D) Cell proliferation assay after *ME1* knockdown. (E) Invasion potential assessed using a transwell assay after *ME1* knockdown.

Our data revealed that *ME1* upregulation was closely associated with poor pathological stage and large tumour size, suggesting that *ME1* contributes to the growth of breast cancer cells (Table 2). We further examined the expression levels of *ME1* in eight breast cancer cell lines, including those with low invasion ability (i.e. MCF7, T47D, SK‐BR3, MDA‐MB‐468 and MDA‐MB‐453) and high invasion ability (i.e. BT549, Hs578T, MDA‐MB‐231 and MDA‐MB‐231‐IV2‐1; Figure 4). We first investigated the biological role of *ME1* in breast cancer cells by employing the knockdown approach. Knockdown of endogenous *ME1* expression was implemented by transfecting the HS578T and MDA‐MB‐231 cell lines with short hairpin (sh) *ME1* and a scrambled control. As presented in Figure 5A, *ME1* expression levels were significantly lower in breast cancer cells transfected with sh*ME1* than in those transfected with a scrambled control. We further investigated the effects of *ME1* on the proliferation, colony formation ability and invasion of breast cancer cells. The colony formation assay revealed that *ME1* knockdown had a significant negative effect on the colony formation ability of the HS578T and MDA‐MB‐231 cell lines (Figure 5B,C). Furthermore, the proliferation of HS578T and MDA‐MB‐231 cells was significantly reduced after *ME1* knockdown (Figure 5D). The invasion ability of HS578T and MDA‐MB‐231 cells was not significantly affected after *ME1* knockdown (Figure 5E). We also examined the biological role of *ME1* in MCF7, which was luminal A subtype. The data indicated that *ME1* knockdown could significantly reduce MCF7 cell growth (Figure 5). The data were consistent with our previous findings indicating that *ME1* expression was positively associated with breast tumour growth and less associated with tumour invasion ability.

### 3.5 *ME1* expression was associated with immune cell infiltration

**FIGURE 6:**
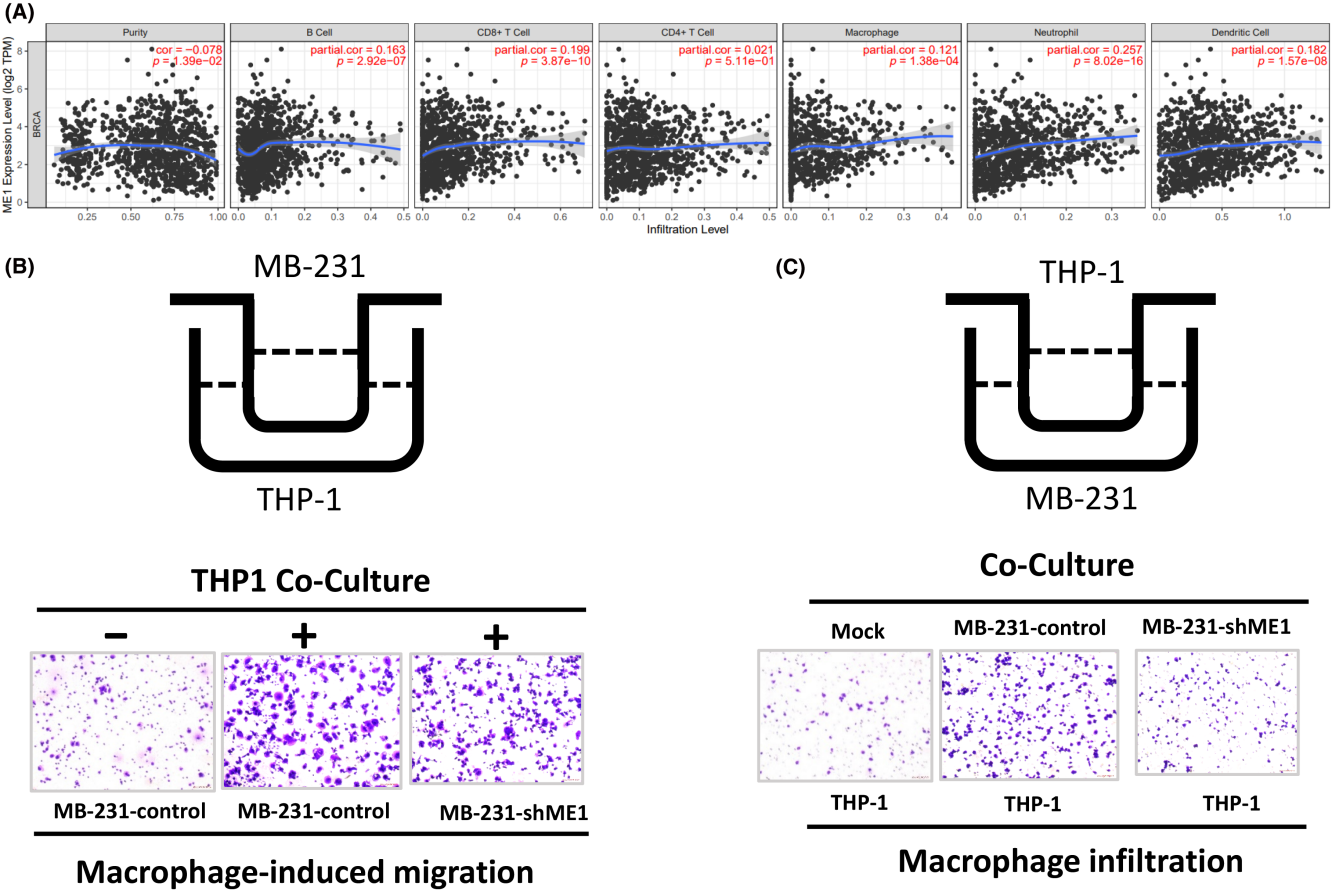
*ME1* genes may influence immune cell infiltration. (A) Correlation between the expression of *ME1* genes and the distribution of immune cells in breast cancer through analysis of the TIMER 2.0 database. (B) Macrophage‐induced migration of breast cancer cells was slightly suppressed in MDA‐MB‐231 cells with *ME1* knockdown. (C) Macrophage infiltration ability was suppressed in MDA‐MB‐231 cells in the *ME1* knockdown control group.

Tumour ecosystems comprise infiltrating immune cells and breast cancer cells, which create a unique tumour microenvironment (TME). However, how *ME1* expression affects the regulation of immune cell infiltration in breast cancer cells is unclear. We analysed the correlation between *ME1* expression and the distribution of immune cells within the TME by employing the Tumor Immune Estimation Resource (TIMER) tool. As presented in Figure 6A, *ME1* expression was significantly and positively correlated with immune cell infiltration status in breast cancer with respect to B cells (r = 0.163, *p* = 2.92E^−7^), CD8+ T cells (r = 0.199, *p* = 3.87E^−10^), macrophages (r = 0.121, *p* = 1.38E^−4^), neutrophils (r = 0.257, *p* = 8.02E^−16^) and dendritic cells (r = 0.182, *p* = 1.57E^−8^). We then investigated the potential interaction between the metastasis of breast cancer cells and the infiltration ability of immune cells. First, we assessed the motility of breast cancer when co‐cultured with macrophages, and the results revealed that macrophages could significantly increase the migration of breast cancer cells. However, the macrophage‐induced migration of MDA‐MB‐231 cells was only mildly affected by *ME1* knockdown (Figure 6B). The macrophage infiltration ability was enhanced when macrophages were co‐cultured with MDA‐MB‐231 cells, whereas it was significantly reduced when macrophages were co‐cultured with MDA‐MB‐231 cells with *ME1* knockdown. Overall, our findings revealed that the poor survival outcomes of patients with high *ME1* expression may be caused by accelerated cancer cell growth and immune cell infiltration.

## 4 DISCUSSION

*ME*s play a critical role in the regulation of NADPH production, and Fan et al. contended that *ME1* produces NADPH at levels comparable to those produced by G6PD, an enzyme in the pentose phosphate pathway.^33^ The Warburg effect has been observed in cancer metabolism.^34^, ^35^, ^36^, ^37^, ^38^ In cancer cells, 90% of glucose is metabolized through aerobic glycolysis, resulting in the production of lactate through fermentation.^39^, ^40^ During aerobic glycolysis, glucose is metabolized into pyruvate and then into lactate. Thus, *ME*s can help produce more pyruvate, which is the main substrate for aerobic glycolysis. Pyruvate is a crucial energy source in cancer cell metabolism^15^, ^34^, ^41^, ^42^; therefore, pyruvate kinase is generally upregulated in cancer cells.^43^ Other glycolytic‐related proteins are generally upregulated in cancer cells, including glucose transporter GLUT1, hexokinase2 and ADP‐dependent glucokinase.^44^ Phosphofructokinase 1 (PFK1) is the rate‐limiting enzyme for glycolysis. PFKFB3‐driven glycolysis is generally observed in cancer cell metabolism. PFKFB3 can generate frucose‐2,6‐biphosphate, an allosteric activator of PFK1. Thus, PFKFB3 activation is also crucial for cancer cell metabolism. In addition, PFKFB3 is generally upregulated in tumour‐associated macrophages (TAMs). This evidence suggests the essential role of glycolysis with upregulated pyruvate in cancer cell metabolism. *ME*s can produce pyruvate and NADPH, both of which are critical for cancer cell survival and invasion. These evidences support our findings regarding why *ME1* upregulation is associated with poor prognosis in patients with breast cancer. In addition, *ME2* can catalyse the chemical reaction from malate to pyruvate. However, *ME2* is an NAD‐dependent mitochondrial *ME*. Unlike *ME1*, *ME2* is in mitochondria, not in cytoplasm. In addition, its substrate is NAD, and it cannot generate NADPH. Thus, *ME2* cannot activate the phagocytosis function in TAMs as well as cancer cells themselves. *ME2* is not as crucial as *ME1* in determining the prognosis of patients with breast cancer. *ME3*, which also catalyses the chemical reaction from malate to pyruvate, is an NADP‐dependent mitochondrial *ME*. Although *ME3* can produce NADPH in mitochondria, it cannot easily generate ROS in the cytoplasm of cancer cells or TAMs to digest environmental tissues or cells. Thus, *ME3* is also less crucial than *ME1* in determining the prognosis of patients with breast cancer. For these reasons, this study focused on the role of *ME1* in breast cancer.

Studies have revealed that *ME1* expression is significantly upregulated in human cancers.^45^, ^46^, ^47^, ^48^ Studies have also observed that tumour‐suppressive micro (mi) RNAs, namely miR‐30a, miR‐30c‐5p, miR‐612 and miR‐885‐5p, could inhibit *ME1* expression by directly targeting 3′UTR of *ME1*.^45^, ^49^, ^50^, ^51^ Furthermore, *ME1* expression levels were regulated by oncogenic transcription factors, including NRF2, β‐catenin and TCF1.^52^, ^53^ In addition, the protein stability of *ME1* was mediated through ERK2‐dependent phosphorylation.^54^ Therefore, the overexpression of *ME1* in breast cancer may result from abnormal transcription factors or miRNA expression.

Biological function assays have revealed that *ME1* knockdown resulted in altered metabolism, reduced cell growth and migration and elevated levels of ROS in human cancer.^15^, ^21^ Liao et al. asserted that *ME1* overexpression resulted from the high amplification ratio of *ME1*. Furthermore, an analysis of a public database (microarray data set) revealed that *ME1* upregulation was significantly associated with large tumour size, advanced tumour grade and poor survival outcomes in patients with breast cancer.^23^ A biological examination revealed that *ME1* expression was involved in glucose uptake and lactate production and reduced oxygen consumption. Furthermore, *ME1* knockdown suppressed tumorigenicity.^23^ Liu et al. reported that *ME1* upregulation was significantly associated with a larger tumour size, a higher incidence of lymph node metastasis and a higher incidence of lymph vascular invasion. *ME1* upregulation was significantly correlated with poorer survival outcomes in patients with breast cancer.^55^ This study data agreed with relevant findings indicating that high *ME1* expression is correlated with advanced clinicopathological stage, tumour size and tumour grade in patients with breast cancer. One study contended that *ME1* tends be upregulated more in TNBC cells than in non‐TNBC cells.^23^ Similar to the results of that study, the results of our IHC analysis also indicated that *ME1* expression was significantly upregulated in TNBC compared with in non‐TNBC (Figure 3E). Notably, we observed that high ER and PR expression were associated with low *ME1* expression, and high HER2 expression was associated with high *ME1* expression (Table 4). A stratification analysis revealed that *ME1* expression was associated with large tumour size and poor tumour grade; however, the results indicated no significant relationship between *ME1* expression and the DSS and DFS of patients with TNBC. In addition, the results did not indicate a relationship between *ME1* expression level and breast cancer metastasis in our cohort. A biological function assay indicated that *ME1* knockdown did not influence breast cancer cell motility in HS578T and MDA‐MB‐231 cells. These findings differ from those of a relevant study.^55^ These inconsistent results may be due to the different genetic backgrounds of the breast cancer cells used in this study as well as the high tumour heterogeneity and different ethnicities of the patients in our breast tissue microarray cohort.

Metabolic dysfunction is generally observed in cancers and in their microenvironments. Metabolism of TAMs is especially crucial in TMEs.^34^, ^56^, ^57^ Glycolysis can be used as an energy generation method in TAMs.^13^ Phagocytosis, the process by which cells digest microorganisms, is a major effector function of macrophages, and this phenomenon can also be observed in TAMs. The metabolism of TAMs is related to the progression of solid tumours, such as breast cancers.^58^, ^59^, ^60^ The characteristics of macrophage metabolism are as follows: First, macrophages express NADPH oxidase enzymes. Macrophages use the pentose phosphate pathway and *ME* pathway to generate NADPH, which enables the production of ROS. These ROS are essential for eliminating digested intracellular macroorganisms, including intracellular bacteria, protozoa and fungi. The pentose phosphate pathway generates NADPH and ribose‐5‐phosphate, which aid in the synthesis of lipids, nucleotides and amino acids, such as histidine. Second, macrophages can use NADPH produced by inducible nitric oxide synthase to generate nitric oxide to eliminate ingested intracellular microorganisms. Third, the NADPH generated through the pentose phosphate pathway and the *ME* pathway can produce antioxidative‐reduced glutathione, which protects macrophages from damage and controls redox homeostasis by counteracting oxidative stress induced by ROS. These characteristics highlight the crucial role of *ME*s in NADPH production, which is essential to the protection and function of macrophages.^33^

Current tumour immunology theories suggest that *M1* macrophages can suppress solid tumours and that *M2* macrophages can promote the growth of solid tumours in TMEs.^59^, ^61^, ^62^, ^63^ However, studies have also suggested that the fusion of tumour cells with macrophages can enable solid tumours to acquire invasion and migration abilities.^64^ Thus, solid tumours can digest additional neighbouring cells or tissues, promoting their growth.^65^, ^66^, ^67^, ^68^ The fusion of solid tumour cells with macrophages also enhances their capacity for distant metastasis. In addition, our previous study asserted that the THαβ antiviral immune response is the primary source of antitumor immunity, whereas the TH1‐like anti‐intracellular microorganism immune response is the primary source of pro‐tumor immunity.^69^, ^70^, ^71^, ^72^, ^73^ Furthermore, TH1 immune reactions with macrophage activation do not exhibit an antitumor immune reaction. Macrophages are the effector immune cells for TH1 and TH1‐like immunity. Studies have revealed that gamma interferon can promote the metastasis of solid tumours.^74^, ^75^, ^76^, ^77^ Gamma interferon can activate *M1* macrophages. Cancer cells fused with macrophages can metastasize to organs in which macrophages are normally present, such as the liver (Kupffer cells), lungs (alveolar macrophages), brain (microglia), bone (osteoclasts) and the pleural space or peritoneum (mesothelial cells). Thus, *M1* macrophages may also promote the invasion and metastasis of solid tumour cells.

Tumour ecosystems comprise infiltrating immune cells, fibroblasts and breast cancer cells, which create a unique TME. The components of TMEs include the extracellular matrix, stromal cells, T cells, B cells, cancer‐associated fibroblasts, TAMs and tumour‐associated neutrophils (TANs).^78^ One study revealed that macrophage infiltration in solid breast tumours is associated with poor prognosis, metastasis and chemotherapy resistance.^58^ This study demonstrated that *ME1* knockdown can inhibit macrophage infiltration. These results suggest that *ME1* upregulation may contribute to poor survival outcomes by altering the TME through its effect on macrophage infiltration.

In summary, our study suggests that *ME1* is related to poor prognosis in patients with breast cancer. This finding can be applied to create a new biomarker of survival outcomes or progression in patients with breast cancer. In addition, new therapeutic agents targeting *ME1* can also be developed against breast cancers and other possible intractable solid tumours.

## AUTHOR CONTRIBUTIONS

**Wan‐Chung Hu:** Conceptualization (equal); formal analysis (equal); investigation (equal); validation (equal); writing – original draft (equal). **Yi‐Fang Yang:** Conceptualization (equal); formal analysis (equal); investigation (equal); validation (equal); writing – original draft (equal). **Ching‐Feng Cheng:** Funding acquisition (supporting); project administration (supporting); resources (supporting); supervision (supporting). **Ya‐Ting Tu:** Data curation (supporting); formal analysis (supporting); methodology (supporting); software (supporting); visualization (supporting). **Hong‐Tai Chang:** Funding acquisition (supporting); project administration (supporting); resources (supporting). **Kuo‐Wang Tsai:** Conceptualization (lead); formal analysis (lead); funding acquisition (lead); investigation (lead); methodology (lead); supervision (lead); validation (lead); writing – review and editing (lead).

## FUNDING INFORMATION

This work was supported by funding from the Ministry of Science and Technology (MOST 111‐2314‐B‐303‐025), Taipei Tzu Chi Hospital and the Buddhist Tzu Chi Medical Foundation (TCRD‐TPE‐MOST‐111‐15 and TCRD‐TPE‐111‐04), and by cooperation between Tzu Chi Hospital and Academia Sinica (TCAS‐112‐02).

## CONFLICT OF INTEREST STATEMENT

The authors declare no conflict of interest.

## CONSENT FOR PUBLICATION

Not applicable.

## Supporting information


Figure S1.

Table S1.

Figure Captions.

## Data Availability

Data available on request from the authors.
