# Supplementary figures and images for: Overexpression of malic enzyme is involved in breast cancer growth and is correlated with poor prognosis
Source: J Cell Mol Med. 2024 Mar 6;28(6):e18163. doi: 10.1111/jcmm.18163 (PMC10915829; doi:10.1111/jcmm.18163)

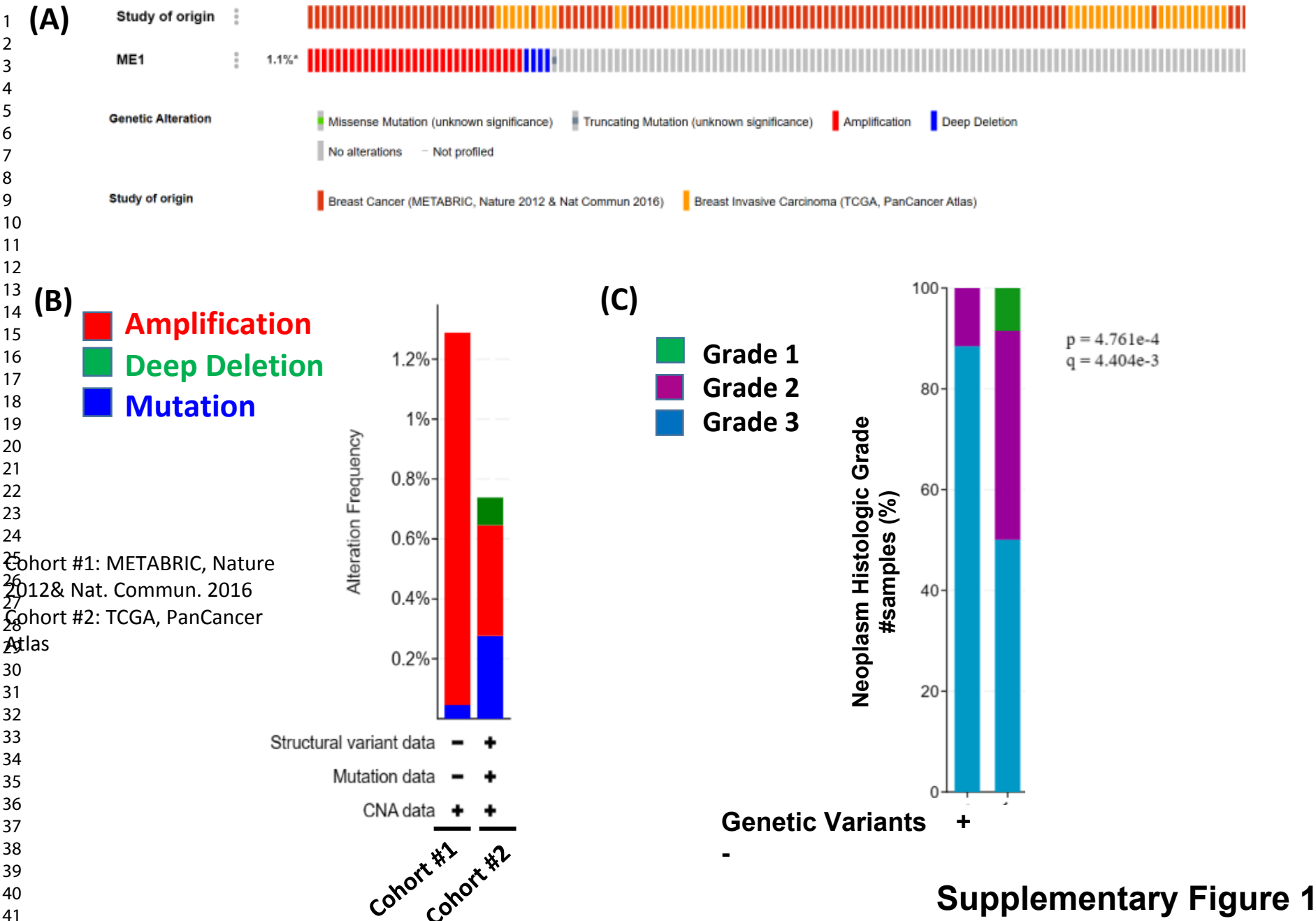

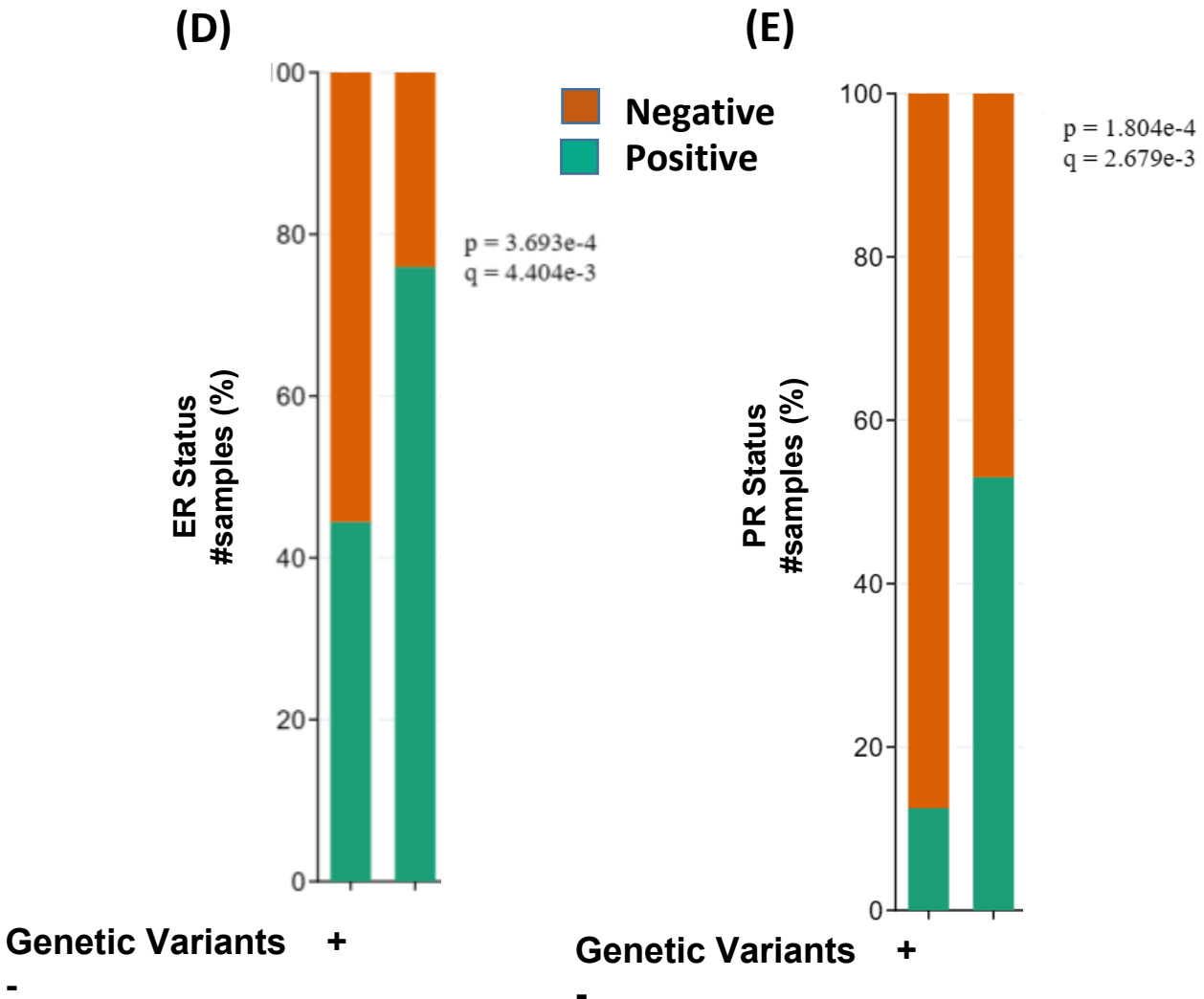

Supplementary Figure 1

(A) GEO database

(B) RNA-Seq

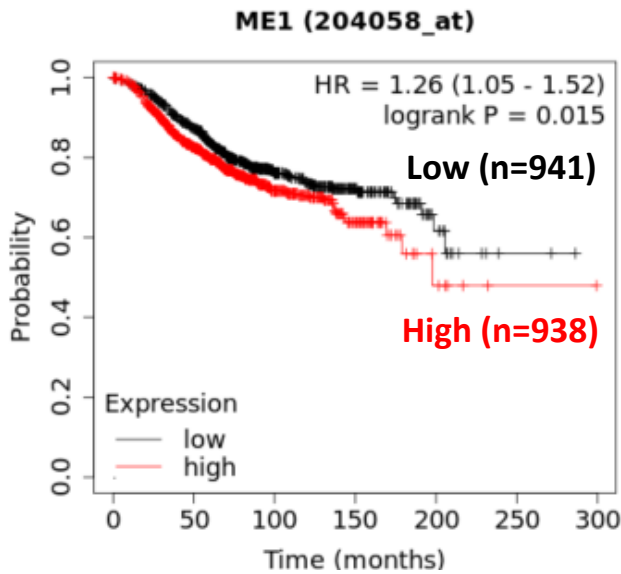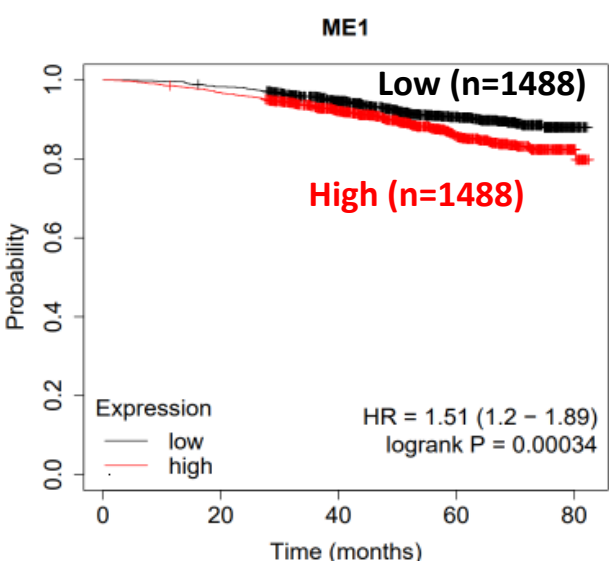

Supplementary Figure 2

Supplement: Supplementary file 1 — Figure S1. [file JCMM-28-e18163-s002.pdf]
