# Supplementary material for: Overexpression of malic enzyme is involved in breast cancer growth and is correlated with poor prognosis
Source: J Cell Mol Med. 2024 Mar 6;28(6):e18163. doi: 10.1111/jcmm.18163 (PMC10915829; doi:10.1111/jcmm.18163)
Supplement: Supplementary file 2 — Table S1. [file JCMM-28-e18163-s003.docx]

| **Supplementary Table 1. Correlation of *ME1* expression with clinicopathological characteristics of 1,079 patients with breast cancer from TCGA** **database** | | | | |
| --- | --- | --- | --- | --- |
| Variables | ME1 (n=1079) | | | |
|  | No. (%) | Mean±SD | Median | p-value |
| **Pathology stage** |  |  |  |  |
| I | 181 (16.8) | 298.02±282.33 | 198.13^de^ | **0.047**^b^ |
| II | 624 (57.8) | 427.74±582.11 | 252.72^d^ |  |
| III | 252 (23.4) | 433.82±861.52 | 201.43 |  |
| IV | 22 (2.0) | 480.76±399.78 | 409.94^e^ |  |
| **pT stage** |  |  |  |  |
| T1 | 274 (25.4) | 359.00±733.43 | 193.38 | 0.142^a^ |
| T2 | 632 (58.6) | 423.43±562.38 | 266.02 |  |
| T3 | 138 (12.8) | 390.26±502.14 | 206.50 |  |
| T4 | 35 (3.2) | 597.78±1010.86 | 283.30 |  |
| **pN stage** (n=1070) |  |  |  |  |
| N0 | 511 (47.8) | 414.82±579.85 | 231.90 | 0.515^a^ |
| N1 | 363 (33.9) | 382.61±457.66 | 232.74 |  |
| N2 | 119 (11.1) | 477.33±1124.09 | 186.01 |  |
| N3 | 77 (7.2) | 379.84±499.16 | 266.38 |  |
| **pM stage** |  |  |  |  |
| M0 | 1057 (98.0) | 406.98±626.40 | 229.84 | 0.582^c^ |
| M1 | 22 (2.0) | 480.76±399.78 | 409.94 |  |
| Sex |  |  |  |  |
| Male | 12 (1.1) | 338.67±332.10 | 243.42 | 0.696^c^ |
| Female | 1067 (98.9) | 409.27±625.11 | 231.88 |  |
| *^a^p-value were estimated by one-way ANOVA test.*  *^b^p-values were estimated by Kruskal-Wallis 1-way ANOVA test.*  *^c^p-value were estimated by student’s T test.*  *^d^P=0.023; ^e^p=0.03* | | | | |

| **Supplementary Table 2. Univariate and multivariate Cox regression analyses of *ME1* expression for the overall survival of patients with breast cancer from TCGA database** | | | | | | |
| --- | --- | --- | --- | --- | --- | --- |
| Characteristic | No. (%) | OS | | | | |
|  |  | CHR (95% CI) | P-value |  | AHR (95% CI) | P-value |
| **ME1** | (n=1079) |  |  |  |  |  |
| Low | 907 (84.1) | 1.00 |  |  | 1.00 |  |
| High | 172 (15.9) | 2.18(1.38-3.43) | 0.001 |  | 2.10 (1.32-3.34) | 0.002 |
| *Abbreviation: DSS, disease****-****specific survival; DFS, disease****-****free survival; CHR, crude hazard ratio; AHR, adjusted hazard ratio*  AHR were adjusted for AJCC pathological stage (II,III and IV VS. I). | | | | | | |

| **Supplementary Table 3. Correlation of *ME1* expression with clinicopathological characteristics of patients with IDC, non-TNBC** | | | | |
| --- | --- | --- | --- | --- |
| Variables | ME1 (n=243) | | | |
|  | % | Mean±SD | Median | p-value |
| Age (yr) |  |  |  |  |
| <40 | 16.0 | 48.16±50.31 | 35.39 | 0.863^a^ |
| 40-59 | 59.3 | 46.73±53.95 | 25.00 |  |
| ≧60 | 24.7 | 51.34±61.25 | 19.91 |  |
| BMI |  |  |  |  |
| Underweight+Normal | 50.6 | 46.34±53.10 | 22.44 | 0.664^b^ |
| Overweight | 28.8 | 55.49±63.39 | 26.60 |  |
| Obesity | 20.6 | 42.07±46.80 | 20.07 |  |
| Menopausal status |  |  |  |  |
| Peri- and pre-menopausal | 47.7 | 50.73±54.99 | 26.28 | 0.478 ^c^ |
| Post-menopausal | 52.3 | 45.69±55.31 | 20.29 |  |
| Pathology stage |  |  |  |  |
| I | 18.9 | 27.92±43.08^de^ | 8.75 | **0.004^b^** |
| II | 47.3 | 55.68±58.41^d^ | 35.54 |  |
| III | 33.7 | 48.78±54.07^e^ | 25.20 |  |
| pT stage |  |  |  |  |
| T1 | 28.4 | 34.23±46.87^fg^ | 12.50 | **0.005^b^** |
| T2 | 63.0 | 50.51±54.26^f^ | 31.59 |  |
| T3+T4 | 8.6 | 76.09±73.18^g^ | 39.68 |  |
| pN stage |  |  |  |  |
| N0 | 42.4 | 45.58±54.20 | 23.20 | 0.834^a^ |
| N1 | 26.7 | 50.26±56.70 | 25.00 |  |
| N2 | 20.6 | 52.84±55.36 | 28.54 |  |
| N3 | 10.3 | 43.36±56.46 | 19.93 |  |
| Grading |  |  |  |  |
| Well+Moderate | 80.7 | 43.25±52.75 | 19.18 | **0.005^c^** |
| Poor | 19.3 | 68.30±60.50 | 50.00 |  |
| Vascular invasion |  |  |  |  |
| Absent | 65.0 | 43.93±53.23 | 19.59 | 0.108^c^ |
| Present | 35.0 | 55.84±57.94 | 29.79 |  |
| Nipple invasion |  |  |  |  |
| Absent | 90.1 | 46.16±54.58 | 21.59 | 0.097 ^c^ |
| Present | 9.9 | 65.81±57.81 | 63.65 |  |
| ^a^*p-value* were estimated by one-way ANOVA test.  ^b^*p-value* were estimated by Kruskal-Wallis one-way ANOVA test  ^c^*p-value* were estimated by student’s T test.  ^d^p=0.001, ^e^p=0.005, ^f^p=0.017, ^g^p=0.004 | | | | |

| **Supplementary Table 4. Correlation of *ME1* expression with clinicopathological characteristics of patients with IDC, TNBC** | | | | |
| --- | --- | --- | --- | --- |
| Variables | ME1 (n=247) | | | |
|  | % | Mean±SD | Median | p-value |
| Age (yr) |  |  |  |  |
| <40 | 13.0 | 60.50±45.87 | 58.18 | 0.244^b^ |
| 40-59 | 56.3 | 62.28±52.88 | 54.09 |  |
| ≧60 | 30.8 | 77.71±63.33 | 59.08 |  |
| BMI |  |  |  |  |
| Underweight+Normal | 49.4 | 64.80±53.89 | 57.43 | 0.831^a^ |
| Overweight | 28.7 | 69.85±60.58 | 59.17 |  |
| Obesity | 21.9 | 67.32±54.17 | 56.61 |  |
| Menopausal status |  |  |  |  |
| Peri- and pre-menopausal | 51.0 | 63.25±52.63 | 57.69 | 0.308^c^ |
| Post-menopausal | 49.0 | 70.49±58.85 | 56.68 |  |
| Pathology stage |  |  |  |  |
| I | 19.8 | 50.55±49.01 | 32.40 | 0.074^a^ |
| II | 52.2 | 70.85±59.09 | 59.68 |  |
| III | 27.9 | 70.76±52.38 | 58.22 |  |
| pT stage |  |  |  |  |
| T1 | 30.8 | 51.24±49.97^e^ | 41.99 | **0.013^a^** |
| T2 | 58.3 | 73.62±56.71^e^ | 65.48 |  |
| T3+T4 | 10.9 | 74.22±59.21 | 73.62 |  |
| pN stage |  |  |  |  |
| N0 | 51.4 | 63.38±55.85 | 50.85 | 0.731^a^ |
| N1 | 23.9 | 72.52±60.24 | 59.68 |  |
| N2 | 15.4 | 70.55±52.62 | 62.26 |  |
| N3 | 9.3 | 64.81±50.00 | 57.80 |  |
| Grading |  |  |  |  |
| Well+Moderate | 42.5 | 57.92±59.93 | 34.42 | **0.002^d^** |
| Poor | 57.5 | 73.36±51.72 | 62.76 |  |
| Vascular invasion (n=245) |  |  |  |  |
| Absent | 57.1 | 69.97±58.99 | 57.71 | 0.490^d^ |
| Present | 42.9 | 62.53±51.09 | 56.14 |  |
| Nipple invasion |  |  |  |  |
| Absent | 96.8 | 67.15±55.47 | 57.61 | 0.588^c^ |
| Present | 3.2 | 56.25±67.47 | 22.64 |  |
| ^a^*p-value* were estimated by one-way ANOVA test.  ^b^*p-value* were estimated by Kruskal-Wallis one-way ANOVA test  ^c^*p-value* were estimated by student’s T test.  ^d^*p-value* were estimated by Mann-Whitney U test.  ^e^p=0.017 | | | | |
