# Supplementary material for: Overexpression of malic enzyme is involved in breast cancer growth and is correlated with poor prognosis
Source: J Cell Mol Med. 2024 Mar 6;28(6):e18163. doi: 10.1111/jcmm.18163 (PMC10915829; doi:10.1111/jcmm.18163)
Supplement: Supplementary file 3 — Figure Captions. [file JCMM-28-e18163-s001.docx]

**Supplementary Figure 1. *ME1* amplification in patients with breast cancer.** (A) Oncoprint indicating genetic alterations of *ME1* in patients with breast cancer. Colors below the oncoprint indicate type of genetic alteration (missense, inframe, truncation, amplification, deletion, fusion) and cohort. (B) The percentages of genetic alterations of ME1 were identified from two independent cohort by analyzing cBioPortal For CANCER GENOMICS database. We obtained cohort 1 data (2173 patients) from METABRIC and cohort 2 data (1084 patients) from PanCancer Atlas. (C) *ME1* with genetic alterations were significantly correlated with poor histologic grades. (D) *ME1* with genetic alterations were positively associated with breast tumors with ER-negative expression. (E) *ME1* with genetic alterations were positively associated with breast tumors with PR-negative expression.

**Supplementary Figure 2**. ***ME1* as a potential target for preventing breast cancer progression.** Kaplan–Meier analysis of overall survival, in accordance with mRNA levels of *ME1*. Data were obtained from publicly available breast cancer online datasets, namely, (A) the Gene Expression Omnibus (GEO), and (B) an RNA sequencing database (using Kaplan–Meier Plotter).
